# Relationships between Religion and Two Forms of Homonegativity in Europe—A Multilevel Analysis of Effects of Believing, Belonging and Religious Practice

**DOI:** 10.1371/journal.pone.0133538

**Published:** 2015-08-06

**Authors:** Stefanie Doebler

**Affiliations:** School of Geography, Archaeology and Palaeoecology, Queen’s University Belfast, Belfast, United Kingdom; University of Vienna, AUSTRIA

## Abstract

This paper examines relationships between religion and two forms of homonegativity across 43 European countries using a bivariate response binary logistic multilevel model. The model analyzes effects of religious believing, belonging and practice on two response variables: a) a moral rejection of homosexuality as a practice and b) intolerance toward homosexuals as a group. The findings indicate that both forms of homonegativity are prevalent in Europe. Traditional doctrinal religious believing (belief in a personal God) is positively related to a moral rejection of homosexuality but to a much lesser extent associated with intolerance toward homosexuals as a group. Members of religious denominations are more likely than non-members to reject homosexuality as morally wrong and to reject homosexuals as neighbors. The analysis found significant differences between denominations that are likely context-dependent. Attendance at religious services is positively related to homonegativity in a majority of countries. The findings vary considerably across countries: Religion is more strongly related to homonegativity in Western than in Eastern Europe. In the post-soviet countries homonegativity appears to be largely a secular phenomenon. National contexts of high religiosity, high perceived government corruption, high income inequality and shortcomings in the implementation of gay rights in the countries’ legislations are statistically related to higher levels of both moralistic homonegativity and intolerance toward homosexuals as a group.

## Introduction

Homosexuality has long been subject to religiously infused debate across Europe. In many Eastern European countries homosexuals face blatant persecution, as the recent criminalization of homosexuality and repeated homonegative riots in Russia, Belarus and Ukraine demonstrate. The violent anti-gay protests that followed nearly every gay pride parade that has taken place in an Eastern European country since the early 2000s [1–4] show that intolerance toward homosexuals is widespread in most of Eastern Europe. Although some significant progress regarding the legalization of same-sex partnerships and marriage has been made in countries like Estonia, and Slovenia, where the parliament has only recently voted for the legalization of gay-marriage [5,6], public attitudes may not always reflect these changes.

In Western Europe, too, gays and lesbians are still far from being universally accepted, despite some positive signals like the recent Irish referendum on gay marriage equality, The ongoing struggles of Western Europe’s mainline churches and several Christian-conservative political parties over the legitimacy of gay marriage and the recent anti-gay marriage protests in London, Dublin, Belfast and Paris [7,8] make clear that in Western Europe, too, homosexuals still struggle for their acceptance.

Negative attitudes toward homosexuals have been operationalized in different ways in the literature. Early psychological studies used the term “homophobia” [9–11], but its use has been criticized for interpreting such attitudes as (medical) phobias and for obscuring the implicit discrimination against homosexuals [11,12]. This article follows a more recent convention and operationalizes negative attitudes toward homosexuality and homosexuals as homonegativity [12,13]. This article distinguishes between two forms of homonegativity: moralistic homonegativity refers to attitudes toward homosexuality as a behavior, while intolerance toward homosexuals as a group is a personal rejection of homosexuals based on out-group prejudice [14–17]. Only a few empirical studies so far have made this distinction [18–20]. Yet, distinguishing between the two forms is important, as they are very different attitudes.

When discussing homonegativity, the influence of religion is an important factor. On anti-gay marches throughout Europe, displays of Christian symbols and references to religious sexual morals could be seen frequently [3,8]. It is common knowledge that the doctrines of the three Abrahamic religions contain passages condemning homosexuality as a sin [19,21–23] It is therefore no surprise that religiosity and church membership have been found to be positively related to homonegativity [24–26]. It should be noted that the teachings of Christianity and Islam also contain passages that remind the believer to ‘hate the sin but forgive the sinner’ [19,27] and to love their neighbors [28]. It is therefore plausible that religiosity could be positively related to a moral rejection of homosexual practice, but at the same time be unrelated to intolerance toward homosexuals as a group. Since the classics of sociology [29], religion has been found to be a main supplier of moral values, thus ensuring social cohesion. Others have emphasized the role of religion as a supplier of collective identities [15,30,31]. These different functions of religion may be differently related to the two forms of homonegativity. More importantly, religion is a multidimensional phenomenon consisting of a believing-, a belonging-, and a practice dimension [32–34]. The three dimensions of religiosity could be differently related to homonegativity. The literature on religion and homonegativity is large and goes back to the 1960s [14,35,36]. Although multidimensional concepts of religion have been around since then [32,37]they have rarely been applied to the study of relationships between religion and homonegativity. The majority of the literature found positive links between homonegativity and various measures of religiosity, such as attendance at religious services and self-rated religiosity [26,36–41]. Religiosity is often operationalized via composite scales without paying much attention to its multi-dimensionality.

The believing dimension consists of traditional doctrinal beliefs, non-traditional beliefs and fundamentalism. A number of authors found traditional believers to be more homonegative than non-traditional and non-believers [26,41–43]. While traditional believers stick to the doctrines of their church, non-traditional believers tend to be more liberal and individualized [44]. and are thus more accepting of lifestyles that deviate from traditional doctrines. Studies taking a modernization theory approach have observed processes of liberalization and individualization of religious beliefs [43,45] not only within the secular strands of European societies, but also within Europe’s churches [44,46,47], which according to them led to a rise in tolerance toward homosexuals [45]. Individualized believers can thus be expected to be less homonegative with regards to both forms of homonegativity than traditional believers.

Fundamentalism is defined as an absolute and exclusive truth-claim of one religion over others [48,49] and has repeatedly been found to be a strong predictor of homonegativity [26,41,42,50,51]. Its exclusivity distinguishes fundamentalism from traditional doctrinal beliefs, such as beliefs in God and beliefs about religious teachings.

The second dimension of religiosity is belonging. Religious belonging is often operationalized via denominational affiliation. Studies have shown that members of religious denominations have less liberal attitudes than non-members and are more likely to endorse homonegative views [24,52,53]. Hayes found religious affiliation in general to be related to conservatism and intolerance toward homosexuals, but Protestants not to differ significantly from Catholics when other variables are controlled for [52,54]. Nonetheless, doctrinal differences between denominations could result in differences in their members’ propensity to tolerate homosexuality and homosexuals as a group.

The findings in the literature on denominational effects thus far are inconclusive. Several American studies found Evangelical Protestants to be more intolerant toward homosexuals than Mainline Protestants, Catholics and Jews [20,36,39,48,55,56]. For the European context, Scheepers, Grotenhuis and Slik [53] and Hayes [52] found no significant difference between Catholics and Protestants, rather, having a religious affiliation per se mattered for tolerance toward minorities (Ibd.). Likewise, Scheepers, Gijsberts and Hello [57] found Catholics to be no more prejudiced toward immigrants than members of other denominations. A number of studies [25,43,58,59] found Muslims to be less tolerant of homosexuality than Catholics, Protestants and orthodox Christians. One might thus expect Muslims to be more intolerant toward homosexuals. However, it is questionable whether Muslims really differ from members of other denominations, once differences in education and wealth and country-level contexts of poverty and democratic governance [59] are adjusted for.

Several factors may contribute to why the findings in the literature are not conclusive. The studies used different surveys and the questions measuring homonegativity differ between surveys. More importantly, the studies looked at different national contexts. The US is a very different context from Europe and even within Europe there is great variation across countries. More cross-national studies using a multilevel framework that can pick up variations in homonegativity across and between countries are needed. The robust pattern found in the literature on Europe so far is that those with a religious affiliation seem to be more likely to hold homonegative views than those without a religious affiliation and that Muslims seem to be less tolerant toward homosexuality than members of other denominations.

The third dimension of religiosity is religious practice. The analysis of this paper focuses on religious practice within a religious community, operationalized via attendance at religious services. This has two reasons: First, the majority of the literature concentrates on religious practice in church, mainly using attendance at religious services as a proxy. Using the same operationalization ensures comparability of the results. Secondly, there is a scarcity of measures of other forms of religious practice, such as prayer, meditation and pilgrimage in the surveys available on Europe. The interest of this paper is in active religious practice in the community. Moral community theory states that being involved in a church and interacting with religious peers fosters pro-social attitudes and norm-conformity and discourages deviant behavior [60,61]. According to social capital theory this is due to social network-effects. Individuals who are actively involved in church are exposed to the religious values and attitudes of their peers [62].

In their study of religion in the American context, Purnam and Campbell found active involvement in religious communities to be linked to pro-social values, but also to intolerance toward diverging opinions and lifestyles, homonegativiy in particular [62]. One can thus expect people who actively participate in religious services to be more likely to endorse moralistic homonegativity. However, because America’s and Europe’s monotheistic religions teach to ‘hate the sin but to love the sinner’ it is plausible to expect involvement in church to be unrelated to intolerance toward homosexuals as a group.

Religion exists not only on the individual level, but also creates national contexts that individuals are exposed to. The moral community hypothesis [60,63,64] emphasizes that on the macro-level the morals of religious communities spill over to the non-religious population, influencing the attitudes even of those who are not actively involved a church. Putnam and Campbell found people living in religious communities to be more likely to be intolerant toward lifestyles that diverge from the religious morals of the majority [62]. One could thus hypothesize that populations of highly religious countries show (on average) more moralistic resentment of homosexual practice.

Other national contexts that were often found to be related to higher levels of homonegativity of populations are a low GDP [25,59], deficits in the implementation of gay-rights in the countries’ legal codes [58,65,66,59] and deficits in governance against corruption. Modernization theory [45,67,68] emphasizes socio-economic contexts of wealth and security as drivers of liberalization, especially as regards gender-attitudes. GDP, good governance, and gay-rights implementation are frequently used measures in this literature. The countries’ levels of gay-rights implementation vary greatly across Europe and are likely to influence public attitudes toward lesbians and gays [5,65].

High levels of corruption of countries are highly correlated with a low GDP. Successful anti-corruption governance was found to be associated with higher tolerance toward various out-groups [69]. Similar findings have been reported with regards to income inequality. Relative deprivation theory [69–71] claims that income inequality is related to anti-social attitudes, and homonegativity. The countries’ levels of perceived corruption, income-inequality and levels of gay-rights implementation are important control variables because they have been theorized to influence both individual-level religiosity and public attitudes toward homosexuals [43,71]. According to modernization theory, religion loses its salience as countries modernize, while at the same time values, such as gender norms and sexual morals become more liberal. Including these contextual variables helps to ensure that any statistical relationships found between religion and homonegativity are robust.

At this point we need to acknowledge some limitations of this article. A literature on homonationalism, “pink-washing” and the “European pink agenda” [72–75] contributes insights into the role of nationalist identity-politics. According to this literature, gay-rights and the institutional acknowledgment of queer identities have been instrumentalized by European governance bodies and national governments to create a notion of a tolerant, gay-friendly Western Europe vis-à-vis an intolerant rest [75]. Puar [73] and Ammaturo [72] point out that such utilizations led to the construction of accepted versus not accepted gay and queer identities that are intersected with race—non-white gays being more disadvantaged than white EU-nationals. At the same time in Eastern European countries, such as Russia, Serbia, Ukraine, Lithuania and Latvia, homosexuals have been persecuted, and gay-rights taunted as expressions of a decadent Western ‘other’ by ultraconservative and religious nationalists [76]. The homonationalism literature has contributed important insights from an institutional meso-level perspective, but these theories are difficult to test with survey data. The EVS, although uniquely well-equipped for cross-national comparisons of social attitudes, does not contain suitable questions to measure nationalism or perceptions of European Pink Agenda politics. The data does also not differentiate attitudes toward different gay identities. These factors are likely to influence homonegative attitudes in Europe, but cannot sufficiently be operationalized with the survey data currently available.

This article presents a multilevel analysis of relationships between religion, a moralistic rejection of homosexuality as a behavior and intolerance toward homosexuals as a group across 43 European countries. The differential effects of each religiosity-dimension on the two forms of homonegativity are examined using a bivariate response binary logistic multilevel model [77,78].

Thus, religion effects on both response-variables will be analyzed and compared simultaneously. This approach is preferable to modeling each response separately, because it allows for a direct comparison of the model coefficients, their standard errors and the residual variances across both responses.

The analysis seeks to answer the following questions: How is religion in Europe related to the citizens’ attitudes toward homosexuality as a practice and toward homosexuals as a group? Do the three religiosity-dimensions, believing, belonging and practice differ in their relationships with the two forms of homonegativity? Lastly, how does the national context matter for the citizen’s likelihood of endorsing homonegative attitudes? Are individuals living in highly religious countries more likely to express homonegative views than individuals living in secular countries, when controlling for the countries’ levels of government corruption, income inequality and deficits in the implementation of gay-rights?

## Hypotheses

In accordance with literature on modernization we expect traditional religious believing to be related to moralistic homonegativity, but unrelated to intolerance toward homosexuals as a group.

***H1:** Traditional doctrinal believing in a personal God is positively related to moralistic homonegativity, but statistically unrelated to intolerance toward homosexuals as a group*.

We hypothesize further that individualized unconventional God-beliefs are negatively related to both forms of homonegativity.

***H2:** Belief in a Spirit/Life Force is negatively related to both moralistic homonegativity and intolerance toward homosexuals as a group*.

Regarding fundamentalism we follow a large literature finding positive links with various forms of intolerance including homonegativity targeted at homosexual practice and homosexuals as a group:

***H3:** People who endorse fundamentalist religious truth-claims are more likely than non-fundamentalist (traditional and non-traditional) religious believers and non-believers to express moralistic homonegativity and to be intolerant toward homosexuals as a group*.

In agreement with the literature on religious belonging, we hypothesize that membership in religious denominations is associated with moralistic homonegativity. However, since Christian and Islamic teachings encourage their members to ‘hate the sin but to forgive the sinner’ [27], it is plausible to expect denominational belonging to be unrelated to intolerance toward homosexuals as a group.

***H4:** Members of religious denominations are more likely to express moralistic homonegativity than the religiously unaffiliated*.

Because research on Europe found Muslims on average to be more intolerant toward homosexuality than members of other denominations, we expect that:

***H5:** Muslims are more likely than members of the other three denominations and non-members to express moralistic homonegativity*.

Since none of the other denominations was found to be more homonegative in its teachings than the others, we do not expect Catholics, Protestants, Orthodox and the religiously unaffiliated to differ significantly in their likelihood of endorsing moralistic homonegativity.

***H6:** Denominational belonging is statistically unrelated to intolerance toward homosexuals as a group. Catholics, Protestants, Orthodox, Muslims and the religiously unaffiliated do not differ statistically significantly in their likelihood of rejecting homosexuals as neighbors*.

For religious practice, similar relationships can be expected. As mentioned above, we expect active involvement in church to be related to moralistic homonegativity, but not to intolerance toward homosexuals as a group. The reasoning behind this is that regular churchgoers tend to be more exposed to the teachings both of heteronormative sexual morals and neighborly tolerance and forgiveness of their church than irregular and non-churchgoers.

***H7:** Religious Practice (Attendance at Religious Services) is positively related to moralistic homonegativity, but statistically unrelated to intolerance toward homosexuals as a group*.

Regarding religious contexts, we hypothesize that:

***H8:** People living in highly religious countries (countries with high mean rates of attendance at religious services) are more likely than people living in secular countries to express moralistic homonegativity*.

## Data

Ethics approval for the research of this article was obtained from the University of Manchester, School of Social Sciences Committee on the Ethics of Research on Human Beings.

The findings are based on a secondary analysis of data from the fourth wave of the European Values Study (EVS) [79]. The data are fully anonymized, were analyzed anonymously and are publicly available online from the GESIS Leibnitz Institute for the Social Sciences data archive, Cologne, Germany [80].

The EVS is a high-quality academic survey including 47 European countries. The data are representative samples of each country’s adult populations of 16 years and older and were collected in each country via multi-stage random probability sampling. The net sample size is 1000–1500 respondents per country, except in Northern Cyprus (N = 500 respondents), Northern Ireland (N = 500 respondents), and Iceland (N = 808 respondents). The data were gathered in the years 2008 to 2010 via computer assisted face-to-face interviews (CAPI). A list of the exact fieldwork periods for each country is published on the data supplier’s website [80] and can also be found in their methods report [81].

To allow for a meaningful cross-national comparison of the two forms of homonegativity, the analysis includes only countries in which both questions were asked. Four countries were excluded from the analysis: Italy was dropped, because the data on moralistic homonegativity (‘Homosexuality is never justifiable’) is not available for Italy. The reason for this is, according to the data provider (GESIS) [82], a translational problem rendering the Italian data on this question incompatible with the other countries of the sample (in Italy the question wording “tenere comportamenti omosessuali” differed in its meaning from the question used in the other countries.

Furthermore, the residual diagnostics of the multilevel model found that Azerbaijan, Kosovo and Macedonia are influential outliers. In order to prevent biased estimates due to these outliers, they were dropped from the analysis. All models were tested for outliers and influential cases using the individual-level and the countries’ df-betas and cook’s distances. The analysis is thus based on N = 61, 661respondents living in 43 countries. 6,930 cases had missing values on one or more variables in the models. Cases with missing values were deleted listwise. All variables that were included in the model were tested for multicollinearity.

### Operationalization

#### Response Variables

Moralistic homonegativity [19,20] is measured via the statement ‘homosexuality is never justifiable’. Intolerance toward homosexuals as a group is operationalized via the statement ‘would not like as neighbors: homosexuals’ (binary, 1 = yes, 0 = no). ‘Homosexuality is never justifiable’ is a 10-point scale (10 = homosexuality is never justifiable, 1 = homosexuality is always justifiable) and was re-coded into a binary variable capturing the strongest disapproval-category (1 = homosexuality is never justifiable) versus the rest. This was done in order to ensure full comparability of the model coefficients between the two response variables in the joint bivariate response multilevel model.45% of the respondents fall into the strongest disapproval-category ‘homosexuality is never justifiable’).

#### Independent Variables

Five indicators of individual-level religiosity are included in the model:

Religious believing is operationalized via belief in a personal God, belief in a Spirit/Life Force and fundamentalism. Belief in a personal God is a traditional belief that accords with the doctrines of the three major monotheistic religions in Europe. Belief in a Spirit/Life Force is a more fuzzy modernized and individualized belief [83]. Both beliefs in God are categories of a question on God-beliefs in the European Values Survey (EVS): ‘Which of these statements comes closest to your beliefs?’–‘there is a personal God’,—‘there is some sort of Spirit or Life Force’,—‘I don’t know what to think’,—‘I don’t really believe there is any sort of God, Spirit or Life-Force’. The respondents could only choose one answer. The atheist answer could not be included because in several countries the number of respondents who chose this answer was too small for meaningful comparisons: e.g. in Armenia 35 respondents, in Cyprus six, in Northern Cyprus 20, in Georgia six, in Romania 21, and in Turkey 21 respondents made this statement. Thus, the atheist statement was collapsed with the agnostic statement ‘I don’t know what to think’ to form the reference category of the analysis.

Fundamentalist believing is operationalized via the statement ‘there is only one true religion (binary). The statement ‘there is only one true religion’ is dummy-coded against the reference category ‘other religions have some basic truths as well’ and ‘all great world religions have some truths to offer’.

As measures of religious belonging four dummy variables for the respondent’s denominational affiliation, Catholic, Protestant, Orthodox and Muslim are included in the models and unchurched (having no affiliation) is left out of the models as the reference category. This choice of reference category avoids the problem of empty cells, as for all denominations except unchurched there are several countries, in which less than five percent of the population are members. As to other denominations, the numbers of Jewish (83), Buddhist, Hindu, and other religious minorities are too small to enable meaningful comparisons. Therefore, members of these denominations were summarized into a category ‘other denomination’ and included in the models.

Religious practice is measured by the frequency of attendance at religious services (7-point scale, 1-never, 2-less than once a year, 3-once a year, 4-only on specific holidays, 5-once a month, 6-once a week, 7-more often). This ensures comparability with prior research as church attendance has been a standard measure of religious practice throughout the literature. Moreover, church attendance is an important indicator of active involvement in a moral community [62].

Country-level religiosity is measured by the aggregated mean rate of attendance at religious services per country.

#### Controls

The following controls were included in the model: education (1—tertiary, 2- above primary and below tertiary, 3—primary was left out as the reference category), whether the respondent has experienced long-term unemployment of three months or more, the respondent’s sex (male as the reference category), being right wing on a political left-right scale (1–10) and preference for a strong leader over a democracy (‘a strong leader who does not have to bother with parliament and elections would be good for the country’) as a proxy for authoritarianism. These controls were chosen because all of them are known to be strongly related to homonegativity. A large literature [43,59,84,85] found that people with low education are more likely to endorse homonegative attitudes. Positive relationships between homonegativity, self-description as politically right-wing and authoritarian attitudes are also well-documented [86–90].

Experiences of long-term unemployment were found to be related to heterosexism [91] and intolerance toward homosexuals and other minorities [59,92]. This has been interpreted as a response to experiences of personal frustration and low social status.

The following variables were included as contextual-level controls: The countries’ levels of perceived corruption are operationalized via Transparency International’s Corruption Perceptions Index (CPI) [93]. The countries’ levels of income inequality are operationalized via their Gini-coefficients [94] and the levels of implementation of gay-rights in the countries’ legislations was operationalized via the International Lesbian, Gay, Bisexual, Trans and Intersex Association’s index of [95,5] lesbian- and gay-rights in the World. The index used here is a four-point scale (4—gay marriage is fully recognized, 3—gay-partnership is legally recognized, but not equal to marriage, 2—gay partnership not legally recognized, 1—gay relationships are illegal) and reflects the status in the European countries at the time of the EVS-fieldwork (2008).

Table 1 contains the summary statistics of all variables of the analysis.

**Table 1:** Summary Statistics of the Variables of the Analysis.

Binary Variables:	N	Percent		Min	Max
‘Homosexuality is never justifiable’	61661	45.1		0	1
‘Would not like as neighbors: homosexuals’	60798	37.3		0	1
Catholic	61661	28.5		0	1
Protestant	61661	12.5		0	1
Orthodox	61661	23.0		0	1
Muslim	61661	08.2		0	1
Other Denomination	61661	02.1		0	1
Belief: Personal God	60935	40.6		0	1
Belief: Spirit/Life Force	60935	32.9		0	1
Belief: Don’t know what to believe	61661	15.6		0	1
Belief: Fundamentalism	60609	22.6		0	1
Sex: female	61649	56.2		0	1
Education: tertiary	61130	23.3		0	1
Education: below tertiary, above primary	61130	57.4		0	1
Long-term unemployment	61661	24.0		0	1
Right-Wing	57074	14.8		0	1
‘Strong leader’	60795	34.6		0	1
Continuous Variables:	N	Mean	Std. Dev.	Min	Max
Church Attendance	60970	3.349	1.940	1	7
Country-Mean Church Attendance	61661	3.349	0.848	1.83	5.67
Corruption Perceptions Index (CPI)^1^	61661	5.623	2.153	2	9.30
Gini-Coefficient^2^	61661	31.875	4.622	24.70	43.20
Gay-rights Index^3^	61661	1.705	0.840	0	4

^1^ The original Corruption Perceptions index (CPI) was re-coded, so that high values mean high corruption.

^2^ High Values mean high inequality.

^3^ Gay-Rights Index: 1 = homosexual relationships are illegal, 2 = homosexual relationships are not legally sanctioned, but not legalized, 3 = gay-partnerships are legally recognized, but are not equal to marriage, 4 = gay marriage is fully legally recognized.

## Analysis

The bivariate response binary logistic multilevel model was run in MLwIN [94] using 2^nd^ order penalized quasi-likelihood estimation (PQL). This estimation technique is appropriate for modeling categorical data [96]. Multilevel models are routinely used for data that have a nested structure [78]. The model presented in this paper operationalizes countries as the level-2-units and individual respondents as the level-1-units, thus individuals are nested within countries. The bivariate response model is a variant of the multivariate response multilevel model [97]. Its main advantage over modeling the two response variables (‘homosexuality is never justifiable’ and ‘would not like as neighbors: homosexuals’) separately is that it allows for a direct comparison of relationships with the independent variables between the two responses, while taking the correlation between them into account. Thus, this method yields more accurate results than modeling the two responses separately.

The model was run stepwise, first as a Null-model, M1 includes the religious denominations, M2 includes all individual-level religion variables, M3 includes religion and individual-level controls and M4, the full model includes all controls and country-level variables. In addition, the models were also run including each independent variable separately. These additional models were run to ensure that no effect is hidden away by the controls. They are not presented here, but are made available as supporting information S1 Dataset.

All model steps were run simultaneously for both responses.

The model has the following form:

$$log\;\left(\frac{\rho ij}{1-\rho ij}\right)=\sum_{t=0}^{n}\;t\;\left[Ztij\;\left(\beta 0tj+\;\sum_{q}^{i}\;\beta qtjXqj\right)\right]$$ (1)

$$\text{Yij}=\beta_{0}{\text{z}}_{1\text{ij}+}\beta "1z1ij\;+"\;\beta "2z1ij\;+\;"\;\beta "3z1ij\;+\mu 1j\;z1ij+\mu 2jz2ij"$$ (2)

$$z1ij=\left\{\begin{array}{l} 1\;if\;\prime Homosexuality\;is\;never\;justifiable\prime \\ 0\;if\;\prime Would\;not\;like\;as\;Neighbours:Homosexuals\prime \end{array}\right\},\;z2ij=1-z1ij$$ (3)

$$Var(\mu 1j)\;=\;\sigma^{2}\mu 1,var(\mu 2j)\;=\;\sigma^{2}\;u2,\;Cov(\mu 1j\mu 2j)\;=\;\sigma \mu_{12}$$ (4)

## Results

**Fig 1:**
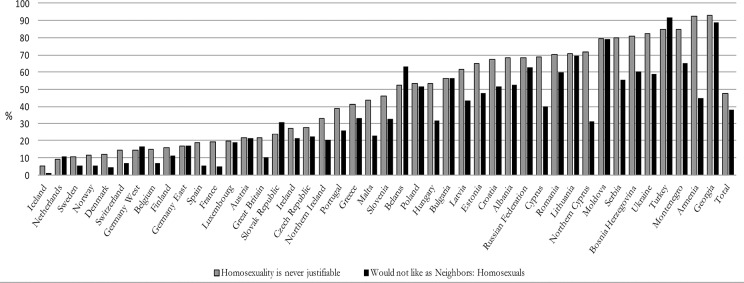
Two Indicators of Homonegativity in Cross-National Comparison. Cross-Country-Percentages, Data: EVS 2008.

The cross-country frequencies of the two response variables in Fig 1 show that homonegativity is a problem in Europe. In half of Europe more than 50% of the population find homosexuality never justifiable and in approximately a Third of the countries more than 50% say they would not like homosexuals as their neighbors. Across the whole sample, 47% of the population find homosexuality never justifiable, 15% chose the two middle categories on the ten-point scale, and only 14% find homosexuality ‘always justifiable’. 37% of Europeans say they would not like homosexuals as their neighbors and 28% gave both homonegative answers. Unsurprisingly, the respondents in most countries are much more reluctant to say they would not like homosexuals as neighbors than to say ‘homosexuality is never justifiable’. Thus, personal resentment toward homosexuals as a group is far less prevalent than a moral rejection of homosexual practice. In cross-European comparison, the Scandinavian and Western European countries are the least intolerant and Eastern and South-Eastern European countries the most intolerant on both indicators.

Table 2 contains the percentages of respondents expressing the two homonegative attitudes across religious categories.

**Table 2:** Percentage of Members of Religious Groups expressing Homonegative Attitudes, Row Percentages.

	‘Homosexuality is never justifiable’, %	‘Would not like as Neighbors: Homosexuals’, %
Catholic	45.0	30.0
Protestant	26.0	13.0
Muslim	86.0	71.0
Orthodox	77.0	58.0
No Affiliation	43.0	30.0
Other Denomination	56.4	33.0
Attends Church once a Month or more often	55.2	45.2
Attend Church less often than once a month	42.7	36.5
Belief: personal God	58.0	48.0
Belief: Spirit/Life Force	39.5	33.0
Belief: Do not know	37.6	34.1
Belief: No Spirit, God or Life Force	29.4	26.0
Belief: Fundamentalism	69.5	57.1

Overall, religiously devout respondents seem more likely to express homonegative attitudes than the non-religious. Fundamentalists are the most likely to express homonegative attitudes, followed by traditional believers, non-traditional believers and non-believers. Orthodox and Muslims are the most prejudiced toward homosexuals and Protestants are the least prejudiced. However, this could be a Scandinavian effect, as the Scandinavian countries all have Protestant majorities. The least homonegative group in terms of religious belonging is unaffiliated (no denomination).

On the practice dimension, people who attend religious services regularly are more likely to endorse homonegative attitudes than those who do not. Obviously, we cannot make causal inferences from uncontrolled raw percentages.

In order to test the hypotheses drawn above, we performed a bivariate response multilevel analysis. Tables 3 and 4 contain the coefficients of the random intercept multilevel model for the two response variables and Table 5 contains the random part of the joint model. The random intercept model fits the coefficients across Europe as a whole, while allowing the intercept to vary across countries. Table 6 contains the random coefficients of each individual-level religion variable and its main effect. These coefficients are obtained by allowing the slope of each religion-variable to vary across countries, rather than assuming that they have the same slope in all countries. Thus, Table 5 gives an overview of the statistical religion effects across Europe as a whole, while Table 6 indicates whether these effects differ statistically significantly between countries. To obtain a clear picture of the between-country variation of the two forms of homonegativity, we first take a look at the Null-model (Tables 2 and 4, M0) and the random part of the joint model (Table 5).

**Table 3:** Bivariate Binary Logistic Multilevel Model–Response 1: Moralistic Homonegativity.

Response 1: Homosexuality is never justifiable’	M0		M1		M2		M3	
	Coef.	S.E.	Coef.	S.E.	Coef.	S.E.	Coef.	S.E.
Fixed Part								
Belief: Personal God			0.175***	0.042	0.263***	0.045	0.273***	0.045
Belief: Spirit/Life Force			-0.259***	0.039	-0.162***	0.042	-0.151***	0.042
Belief: Do not know			-0.023	0.041	0.018	0.044	0.027	0.044
Belief: Fundamentalism			0.660***	0.026	0.600***	0.028	0.603***	0.029
Catholic			0.129***	0.038	0.089*	0.039	0.105**	0.039
Protestant			0.106*	0.050	0.088	0.050	0.107**	0.050
Orthodox			0.016	0.043	0.028	0.042	0.029	0.043
Muslim			0.766***	0.069	0.709***	0.070	0.707***	0.069
Other Denomination			0.568***	0.079	0.577***	0.080	0.587***	0.080
Attendance at Religious Services			0.106***	0.007	0.108***	0.007	0.108***	0.007
Education: tertiary					-0.808***	0.035	-0.812***	0.035
Education: below tertiary, above primary,					-0.338***	0.030	-0.340***	0.030
Sex: female					-0.426***	0.022	-0.428***	0.022
Long-term unemployment					-0.097***	0.027	-0.100***	0.027
Right-Wing					0.307***	0.030	0.308***	0.030
Strong Leader					0.176***	0.023	0.176***	0.023
Country-Mean Attendance at Religious Services							0.362***	0.081
Corruption (CPI)							0.338***	0.069
Gini-Coefficient							0.050***	0.024
Gay-rights Implementation							-0.539***	0.172
Constant	-0.323	0.198	-0.5	0.186	-0.041	0.194	0.023	0.111

* P<0.05

** P<0.01

*** P<0.001

Reference categories of the categorical variables in the model: believing: ‘no God/Spirit/Life Force’ & ‘not stated’, belonging: ‘no affiliation’, education: primary and below primary education, sex: male, right-wing: not being right-wing, strong leader: no strong leader.

**Table 4:** Bivariate Binary Logistic Multilevel Model–Response 2: Intolerance toward Homosexuals as a Group.

Response 2: Would not like as Neighbors: Homosexuals’	M0		M1		M2		M3	
	Coef.	S.E.	Coef.	S.E.	Coef.	S.E.	Coef.	S.E.
Fixed Part								
Belief: personal God			-0.012	0.007	-0.002	0.007	-0.001	0.007
Belief: Spirit/Life Force			-0.047***	0.006	-0.032***	0.007	-0.031***	0.007
Belief: Do not know			0.004	0.007	0.009	0.007	0.010	0.007
Belief: Fundamentalism			0.066***	0.005	0.054***	0.005	0.054***	0.005
Catholic			0.011	0.006	0.010	0.006	0.011	0.006
Protestant			0.007	0.008	0.006	0.008	0.009	0.008
Orthodox			0.028**	0.008	0.030***	0.008	0.028***	0.008
Muslim			0.064***	0.012	0.054***	0.012	0.052***	0.012
Other Denomination			-0.006	0.013	-0.006	0.013	-0.005	0.013
Attendance at Religious Services			0.010***	0.001	0.011***	0.001	0.011***	0.001
Education: tertiary					-0.081***	0.006	-0.081***	0.006
Education: above primary, below tertiary					-0.037***	0.005	-0.037***	0.005
Sex: female					-0.051***	0.004	-0.052***	0.004
Long-term unemployment					0.011*	0.004	0.011*	0.004
Right-Wing					0.042***	0.005	0.042***	0.005
Strong Leader					0.028***	0.004	0.028***	0.004
Country-Mean Attendance at Religious Services							0.027*	0.012
Corruption (CPI)							0.058***	0.011
Gini-Coefficient							0.012**	0.004
Gay-rights Implementation							-0.078**	0.028
Constant	0.354	0.036	0.345	0.034	0.388	0.034	0.400	0.018

* P<0.05

** P<0.01

*** P<0.001

Reference categories of the categorical variables in the model: believing: ‘no God/Spirit/Life Force’ & ‘not stated’, belonging: ‘no affiliation’, education: primary and below primary education, sex: male, right-wing: not being right-wing, strong leader: no strong leader.

**Table 5:** Shared Random Part of the Bivariate Response Multilevel Model.

	M0		M1		M2		M3	
*Random Part* Level: Country		S.E.		S.E.		S.E.		S.E.
Sigma-squared v0: Level-2 Variance of response 1	1.678	0.363	1.423	0.308	1.521	0.329	0.425	0.093
Sigma-squared v0: Level-2 Variance of response 2	0.276	0.063	0.229	0.054	0.231	0.054	0.032	0.012
Aggregate Correlation between response 1 and response 2	0.891		0.864		0.858		0.460	
Individual-level Corelation between response 1 and response 2	0.234		0.219		0.208		0.208	
-2*Log-likelihood			120163		105773		105623	
AIC			120185		105805		105664	
BIC			120284		105947		105841	
Countries	43		43		43		43	
N	61661		59653		54731		54731	

* P<0.05

** P<0.01

*** P<0.001.

**Table 6:** Random Slope Effects of the Individual-Level Religion Measures of the Multilevel Model.

Random Slope Effects of Individual-Level Religion Variables	Response 1: 'Homosexuality is never justifiable'		Response 2: 'Would not like as Neighbors: Homosexuals'	
	Random Slope Variance	S.E.	Random Slope Variance	S.E.
Believing:				
Belief: Personal God, Main Effect	0.223*	0.111	-0.020	0.053
Belief: Personal God, Random Slope	0.480***	0.107	0.066**	0.020
Belief: Spirit/Life Force, Main Effect	-0.206*	0.102	-0.181***	0.053
Belief: Spirit/Life Force, Random Slope	0.396***	0.090	0.075**	0.022
Belief: Fundamentalism, Main Effect	0.713***	0.144	0.326***	0.082
Belief: Fundamentalism, Random Slope	0.846***	0.191	0.230***	0.059
Belonging:				
Catholic: Main Effect	0.040	0.076	0.074	0.055
Catholic: Random Slope	0.114**	0.041	0.046*	0.020
Protestant: Main Effect	0.180**	0.088	0.082	0.088
Protestant: Random Slope	0.081	0.043	0.123*	0.054
Orthodox: Main Effect	0.264	0.195	0.310**	0.130
Orthodox: Random Slope	0.814***	0.267	0.310**	0.116
Muslim: Main Effect	0.814*	0.305	0.300	0.228
Muslim: Random Slope	2.077**	0.705	0.897**	0.358
Practice:				
Attendance at Religious Services, Main Effect	0.096***	0.020	0.055***	0.011
Attendance at Religious Services, Random Slope	0.015***	0.004	0.003**	0.001

* P<0.05

** P<0.01

*** P<0.001

Reference categories of the categorical variables in the model: believing: ‘no God/Spirit/Life Force’ & ‘not stated’, belonging: ‘no affiliation’, education: primary and below primary education, sex: male, right-wing: not being right-wing, strong leader: no strong leader.

The Null-model does not include covariates. The intercepts in Tables 3 and 4 (M0) reflect the average agreement to the two responses, which differs greatly between the two forms of homonegativity. The between-country variances of the Null-model in Table 5 show that endorsement of moralistic homonegativity varies more between countries than intolerance toward homosexuals as a group.

Table 5 contains the between-country variances of the two response variables, the aggregate-level and individual-level correlations between the two responses, the -2-log likelihood and the model fit statistics AIC and BIC of the joint bivariate model. Looking at Table 5, we see that the unexplained level-2 variances of both responses are reduced considerably with the inclusion of the controls, most strongly with the inclusion of the country-level indicators in M3. For response 1 (‘homosexuality is never justifiable’) the unexplained between-country variance is reduced from 1.678 in M0 to 0.425 in M4, and for response 2 (‘would not like as neighbors: homosexuals’) the between-country variance reduces from 0.231 to 0.032 between M0 and M3. This tells us that the explanatory variables explain a very satisfactory part of the variation in homonegativity. The likelihood-ratio tests, AIC and BIC values all indicate a clear and statistically significant model fit improvement for each step of the model, especially for the final model (M4).

Regarding the believing dimension, we hypothesized in H1 that traditional doctrinal believing in a personal God is positively related to moralistic homonegativity, but statistically unrelated to intolerance toward homosexuals as a group. We hypothesized further in H2, that individualized, unconventional God-beliefs are negatively related to both forms of homonegativity.

The model coefficients in Tables 3 and 4 show that traditional doctrinal belief in a personal God is indeed positively related to moralistic homonegativity, but is not statistically significantly related to intolerance toward homosexuals as a group. Belief in a Spirit/Life Force, on the other hand is strongly negatively related to both forms of homonegativity. The findings thus support H1 and H2. Furthermore, as hypothesized in H3, belief in the fundamentalist religious truth-claim ‘there is only one true religion’ is strongly positively related to both moralistic homonegativity and intolerance toward homosexuals as a group.

**Fig 2:**
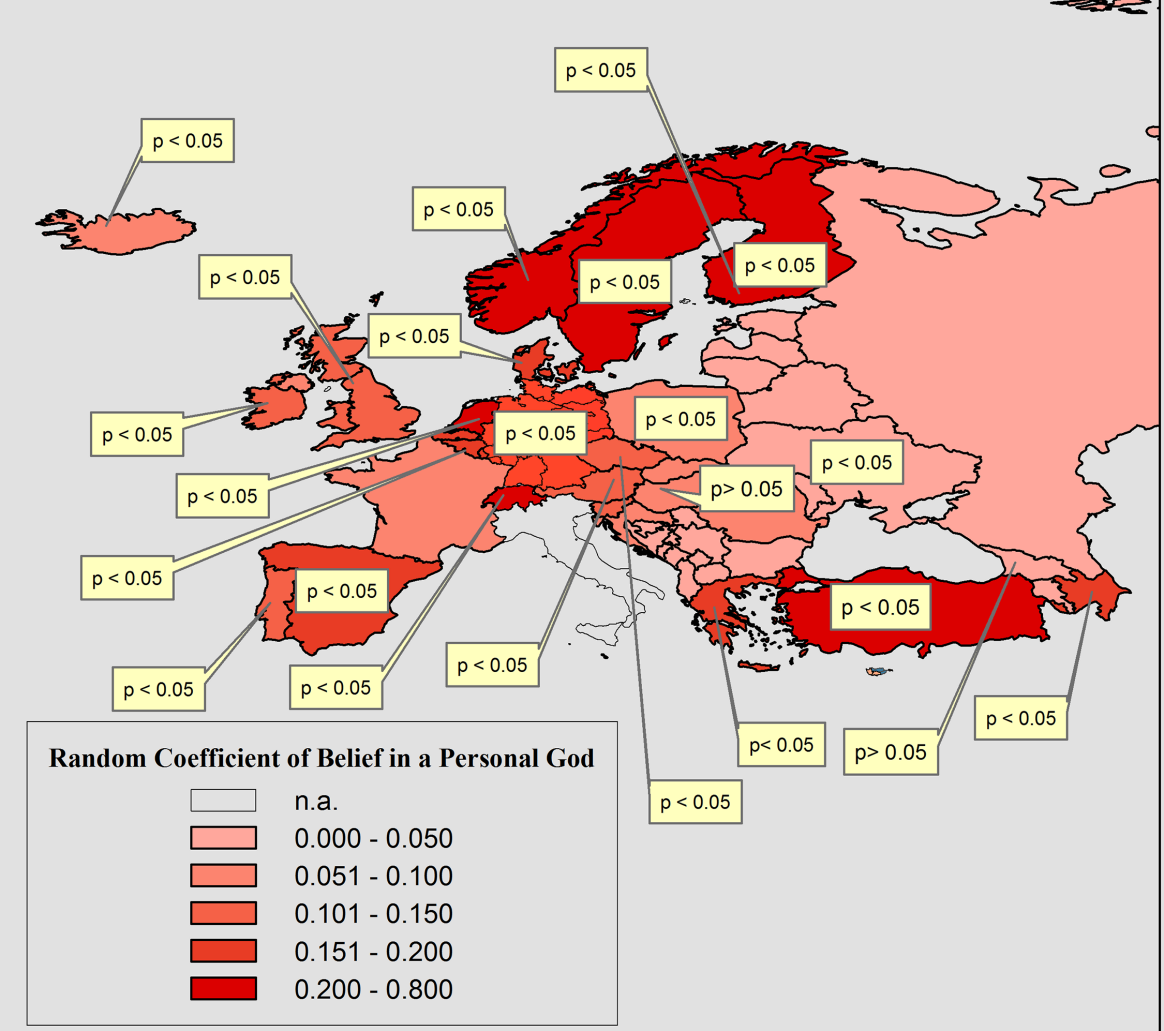
The Variation of the Statistical Effect of Belief in a Personal God on Moralistic Homonegativity (‘Homosexuality is never justifiable’). In Textboxes: the p-value (<0.05) of countries which showed a statistically significant random coefficient; n.a.: question was not asked.

**Fig 3:**
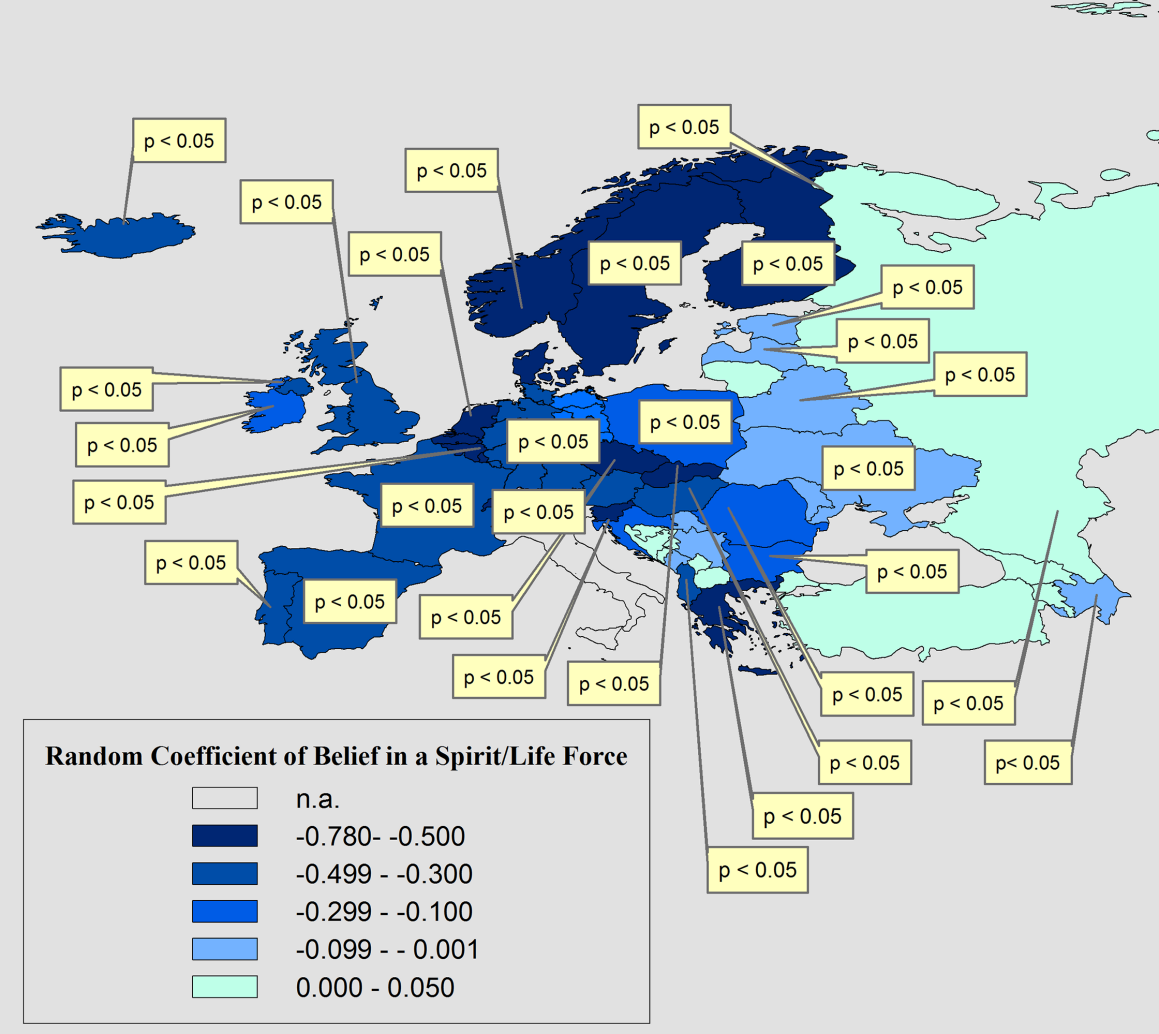
The Variation of the Statistical Effect of Belief in a Spirit/Life Force on Moralistic Homonegativity (‘Homosexuality is never justifiable’). In Textboxes: the p-value (<0.05) of countries which showed a statistically significant random coefficient; n.a.: question was not asked.

Interestingly, when looking at the random coefficients of religious believing in Table 6, we see that they all vary significantly between countries. Fig 2 and Fig 3 visualize the pattern of the variation of the coefficients of belief in a Personal God and Belief in a Spirit/Life Force across countries for moralistic homonegativity. From Fig 2 and Fig 3 we see clearly that the cross-country pattern of the coefficients follows a divide between Western Europe versus the post-communist countries of Eastern- and South-Eastern Europe. The statistical effects of both beliefs in God and of fundamentalism (not shown here) are consistently stronger and statistically significant in Western European countries, but much weaker in the East and South-East. In many Eastern European countries they are not statistically significant at all.

The random coefficient variation shows a very similar, but weaker pattern for the response ‘would not like as neighbors: homosexuals’. Also, fundamentalist believing shows a very similar cross-country pattern as belief in a Personal God, except for the larger effect sizes (coefficients). Because of the very similar pattern, maps of the effects of these variables are not supplied here. Their random coefficients and standard errors are displayed in Table 6.

Regarding the belonging-dimension, we hypothesized in H4 that members of religious denominations are more likely than people with no affiliation to express moralistic homonegativity. In H5 we hypothesized that Muslims are more likely than non-Muslims to express moralistic homonegativity and in H6 we hypothesized that denominational belonging is statistically unrelated to intolerance toward homosexuals as a group.

Looking at the model coefficients across the two response variables in Tables 3 and 4, we see clear denominational differences. Members of all religious denominations, Muslims in particular are more likely than non-members to express one or both homonegative attitudes. The higher likelihood of Muslims of expressing homonegativity concurs with findings in some of the literature [25,43]. When holding religious belief and practice constant, Orthodox members are no more likely than people with no religion to reject homosexuality as morally wrong, but they are the second most homonegative group when it comes to rejecting homosexuals as neighbors. Among the Orthodox, it is the more devout members that tend to express a moral resentment against homosexuality. Members of the other three denominations tend to hold a moral resentment regardless of their levels of devoutness and belief.

When it comes to rejecting homosexuals as neighbors, thereby expressing a strong social distance toward them, both Orthodox and Muslims stand out as the most intolerant denominations independent of their levels of religious practice and belief, while Catholics and Protestants are no more likely than people with no affiliation to reject homosexuals. This difference between denominations is robust when controlling for religious, political and economic national contexts.

The random coefficients of denominational belonging in Table 6 show a statistically significant between-country variation. This is not surprising, as it is known that denominations are highly clustered by countries. E.g. 84% of the Orthodox and 59% of the Muslim population in our sample live in an Eastern European country, another 37% of the Muslims live in Southern Europe and almost 60% of the Catholics live in South-Eastern Europe. This clustering is reflected in the results.

We can summarize that H4 and H5 are supported by the data, while H6 is not. The analysis did find significant differences between denominations and found denominational membership to be statistically positively related not only to moralistic homonegativity, but also intolerance toward homosexuals as a group.

Concerning religious practice we hypothesized in H7 that attendance at religious services is positively related to moralistic homonegativity, but statistically unrelated to intolerance toward homosexuals as a group. Tables 3 and 4 make clear that attendance at religious services is statistically significantly positively related to both forms of homonegativity and the effect holds when including the controls. H7 is thus only partly confirmed. Religious practice in church is related not only to a moral rejection of homosexual behavior, but also to strong social distance toward homosexuals.

**Fig 4:**
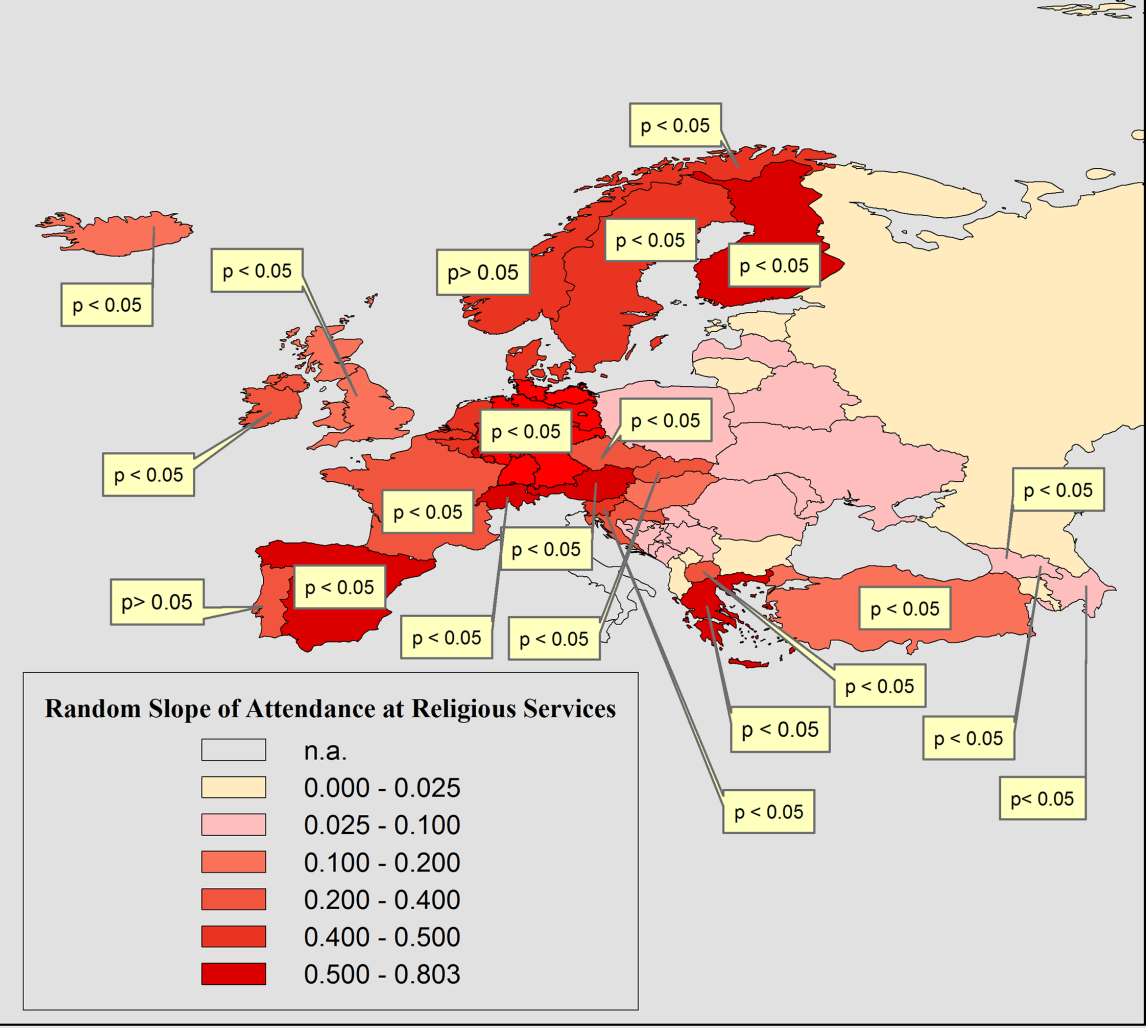
The Variation of the Statistical Effect of Attendance at Religious Services on Moralistic Homonegativity (‘Homosexuality is never justifiable’). In Textboxes: the p-value (<0.05) of countries which showed a statistically significant random coefficient; n.a.: question was not asked.

As with the other religiosity-measures, we allowed the slope of attendance at religious services to vary across countries. The random slope variance for both homonegative responses is shown in Table 6. In order to make it more visually intuitive, the random slope of attendance at religious services on moralistic homonegativity was mapped across countries in Fig 4. The same pattern was also observed for ‘would not like as neighbors: homosexuals’ as the response (not displayed here).

Fig 4 shows that the coefficients of attendance at religious services follow a West-East-divide: The relationships with the two homonegative responses are stronger in Western- than in Eastern Europe. In most Eastern European countries the coefficient is not statistically significant at all. We thus observe the same pattern for the practice dimension as seen for the believing dimension of religion. toward homosexuals as a group.

Table 7 contains chi-squared difference tests of each model coefficient across the joint bivariate response model. The chi-squared difference tests were carried out for each coefficient separately. They test whether the difference in the coefficient of each variable across the two responses equals zero. Thus, a statistically significant test of an independent variable means that its coefficient differs significantly between the two response variables. The chi-squared tests in Table 7 indicate that the differences in the size of the religion-coefficients between the two forms of homonegativity are all statistically significant.

**Table 7:** Chi-Squared Difference Tests of the Joint Model Coefficients across the Two Response Variables.

Independent Variable	Wald	Df.
Catholic	8.77*	(2df)
Protestant	9.48*	(2df)
Orthodox	18.30***	(2df)
Muslim	95.57***	(2df)
Church Attendance	274.66***	(2df)
Belief: Personal God	39.45***	(2df)
Belief: Spirit/Life Force	28.63***	(2df)
Belief: Fundamentalism	493.65***	(2df)
Country-Mean Church Attendance	20.85***	(2df)
Corruption (CPI)	34.95***	(2df)
Gini-Coefficient	11.15**	(2df)
Gay-rights implementation	12.25**	(2df)

Note: The degrees of freedom are shown in parentheses.

* P<0.05

** P<0.01

*** P<0.001.

The last hypothesis refers to the national context.H8 hypothesized that living in countries with high mean rates of attendance at religious services is positively related to moralistic homonegativity. Model M4 shows that country-level religiosity (the countries’ mean rate of attendance at religious services) is statistically positively related to both forms of homonegativity. The higher a country’s average level of religiosity, the higher is the proportion of individuals endorsing one or both homonegative attitudes among its population. The religious moral community in Europe may foster trust and good neighborliness [98,99], but according to the EVS-data, at least on the country-level it is also strongly associated with intolerance toward homosexual lifestyles and toward homosexuals. H8 is therefore confirmed by the data.

The coefficients of the country-level controls are as expected based on the literature: High levels of perceived government corruption (CPI) and income inequality of countries are strongly significantly positively related to both moralistic homonegativity and intolerance toward homosexuals. A high degree of gay-rights implementation in a country, on the other hand, is negatively related to its citizens’ likelihood of endorsing homonegative attitudes.

The country-level variances of the two response variables, as well as the correlation between them decrease considerably and statistically significantly when the country-level variables are included. From M3 to M4 (Table 5) the between-country variance of ‘homosexuality is never justifiable’ decreases from 1.521 to 0.425 and the between-country variance of ‘would not like as neighbors: homosexuals decreases from 0.231 to 0.032. Thus, the countries’ levels of religiosity, corruption, income inequality and the degree of gay-rights implementation explain 72% of the between-country variation of ‘homosexuality is never justifiable’ and 86% of the between-country variation of ‘would not like as neighbors: homosexuals’. Thus, the contextual variables capture the between-country differences both forms of homonegativity very well.

## Discussion

The analysis of this paper tried to disentangle relationships between religious believing, belonging and practice and two forms of homonegativity in Europe. The findings reveal interesting patterns: Of the two forms of homonegativity, a moral rejection of homosexual practice has the higher average endorsement-rates across countries, but also the greater cross- country variation. Both religious and non-religious Europeans are more reluctant to say they would not like homosexuals as neighbors than to express a moral rejection of homosexual practice. The finding makes sense. In contrast to a mere disagreement on sexual morals, a rejection of homosexuals as neighbors refers to them on a personal level and is therefore the stronger measure of social distance.

As hypothesized in H1, we find that traditional doctrinal believing is statistically positively related to moralistic homonegativity, but unrelated to intolerance toward homosexuals as a group. The finding makes sense, as traditional doctrinal believers identify with the teachings of their churches, which in the case of Europe’s majority denominations condemn homosexuality as a sin, but at the same time encourage neighborliness and forgiveness. The finding confirms that morally rejecting homosexual behavior and intolerance toward homosexuals as a group are qualitatively different forms of homonegativity. This difference is clearly reflected in the data. The notion that the religious teaching to ‘hate the sin, but to love the sinner’ may prevent traditional believers from rejecting homosexuals on a personal level is supported by our findings. Those who endorse the fundamentalist truth-claim ‘there is only one true religion’, on the other hand, are significantly more likely to express both moralistic homonegativity and intolerance toward homosexuals as a group. The finding supports H3 and likely reflects a generally more intolerant mindset of fundamentalists. It is plausible that people, who are not open to the possibility that other religions may also have some valid truth to offer, are also less likely to tolerate divergent lifestyles, such as homosexuality. The exclusivity of the fundamentalist truth-claim likely encourages feelings of social distance toward moral out-groups. As mentioned above, the statement ‘would not like as neighbors: homosexuals’ is a measure of social distance [100,101] is plausible that those, who seek to isolate themselves from diverging beliefs, tend to also seek physical distance to those, whose lifestyles they disapprove of. Modern, individualized belief in a Spirit/Life Force, on the other hand is strongly negatively related to both forms of homonegativity. The findings hold for most of Europe except the post-soviet Eastern European countries.

Table 6 showed that when allowing the slopes of believing to vary across countries their main effects remain statistically significant across Europe as a whole. The findings thus confirm H1, H2 and H3 for the majority of countries. They accord with modernization theory’s distinction between traditionalist and liberal attitudes [45,102] and tie in with a literature finding different images of God to be differently related to intolerance toward homosexuals [103–105].

However, the fact that religious believing matters more in Western, Northern and Southern Europe while in most of the (less modernized) Eastern European countries it makes no statistically significant difference to people’s attitudes toward homosexuals also support approaches that are critical of modernization theory. Hervieu-Legér [106], Emerson and Hartman [107], and Clairmont and Browning [108] remind us that religious traditionalism and intolerant attitudes can be cultural responses to modernization itself (not just a lack of it) and can thus have significance within modernized settings.

Furthermore, the findings lend some support to theories of Pink-washing, and homonationalism [72,73,75]. The distinction between traditionalist, liberal-individualized religious believers and non-believers with regards to their attitudes toward homosexuals is greatest in precisely the countries that are governed by what a recent literature described as a Western European homonationalist “Pink Agenda” [72,73] of policies defining the acceptance and legal protection of homosexual identities as a condition of membership in a “free and tolerant Europe” [72]. In Eastern Europe, on the other hand, where homonegative discourses are highly prevalent, propagated by political leaders and inscribed in the legislations of countries like Russia, Ukraine and Serbia religious believing has little statistical significance. Homonegativity in Eastern Europe is not driven by traditionalist religious beliefs, but is a widely socially accepted phenomenon, often seen as part of a nationalist resistance against values that are seen as foreign and imposed [76] by a “decadent West”. The results make clear that it is important not just to look at the individual level, but to contextualize the findings. The between-country variation in the effects of religious believing is large and follows a clear post-communist-Eastern-versus-the-rest-of-Europe—divide.

Looking at religious belonging, members of all denominations are more likely than people with no religious affiliation to express both forms of homonegativity. This finding is surprising. Given that the teachings of both Christianity and Islam encourage members to love their neighbors and to forgive those considered sinners [27], one would expect denominational affiliation to be related to moralistic homonegativity, but not intolerance toward homosexuals as a group. Muslims in particular stand out as the most likely to morally condemn homosexual behavior. Both Muslims and Orthodox are also more likely than members of the other denominations and non-members to reject homosexuals as neighbors. We can thus summarize that H4 and H5 are confirmed by the data, while H6 is not supported.

As was the case with religious believing, the strong variation between countries shows how important it is to contextualize the results and to avoid hasty generalizations based solely on individual coefficients. Denominational membership is highly clustered within countries. 84% of the Orthodox and 80% of the Muslims in the sample live in Eastern and South-Eastern Europe. Many countries in this region still suffer deficits in socio-economic modernization and governance. However, the models controlled for modernization measures.

Other plausible contextual factors are the countries’ historical legacies [109]. The vast majority of Eastern Europe and large parts of the South-East, have legacies of communist rule and of ethnic and religious conflict. In Russia, Belarus, Ukraine, Serbia, Albania, Armenia, Latvia [22], Lithuania and Turkey nationalist rhetoric by political and religious leaders [110–112] is known to have been intertwined with homonegative discourses for decades. At the same time, Orthodoxy and Islam have been defined by political elites as counter-identities vis-à-vis “the West” and against local cultural out-groups. Thus, the higher likelihood of Muslims and Orthodox of endorsing homonegative attitudes is likely due to the strong role of religious affiliation as a national identity marker in these countries. The interplay of collective group-identification and out-group intolerance was described by social identity theory [15,113].

Identity theory claims that such contexts foster perceptions of group-threat and anti-out-group attitudes [15,114]. To sum up, it is unlikely that there should be something intrinsic to the teachings of Islam and Orthodoxy rendering their members more homonegative than members of other monotheistic religions. The most likely drivers of the correlation found between Orthodox and Muslim belonging and homonegativity are nationalism and the intervention of political and religious elites preaching bigotry in several regions of Eastern and South-Eastern Europe.

However, the import of such cultural contexts is difficult to operationalize with survey data. It does not make analytical sense to include dummy indicators for these contexts into our model, as these would include most of South-Eastern and Eastern Europe, which is almost half the sample. Such measures would therefore not discriminate sufficiently for a meaningful statistical analysis. Furthermore, we cannot operationalize the explanatory power of cultural discourses and ideologies with the EVS-data, as we do not have appropriate measures in the survey. These are limitations of this paper. With regard to religious practice, we found that attendance at religious services is positively related to both forms of homonegativity. This contradicts H7, which hypothesized a positive relationship only with moralistic homonegativity. The reasoning was that those who attend religious services are more exposed to teachings of neighborly love and forgiveness, and thus less intolerant toward homosexuals, but this is not supported by the data. On the other hand, the scriptures of Europe’s monotheistic religions contain numerous counter-examples of intolerance. Religion has both the potential to promote neighborliness and forgiveness and to foster bigotry. Religious leaders can choose to preach one or the other. Whether involvement in church fosters tolerance or intolerance likely depends on the context, on how religious leaders teach their religion and on peer-group dynamics within religious communities.

Fig 4 showed that the statistical effect of religious attendance varies greatly between countries. As was the case with religious believing and belonging, we found a clear West-East-divide: Attendance at religious services shows strongly significantly positive relationships with moralistic homonegativity in Western, Northern and Southern Europe, but not in the post-soviet countries of Eastern Europe.

In summary, traditional individual-level religiosity is positively related to homonegativity in most of Europe, except the post-soviet East, where homonegativity appears to be largely a secular phenomenon. The high levels of homonegativity in Eastern Europe, on the other hand, are likely a response to homonegative and anti-Western discourses that prevail in many Eastern European countries. These discourses are largely secular. The only religiosity dimension that plays to these discourses seems to be religious belonging.

The last step of the analysis tested for the statistical effect of the countries’ mean rates of attendance at religious services, controlling for perceived corruption, gay-rights implementation and income inequality. The analysis showed that the countries’ mean rate of attendance at religious services is statistically significantly positively related to both forms of homonegativity.

The finding that people living in highly religious countries are more likely than people in secular countries to morally reject homosexuality is no surprise. According to theory [60,63] religious communities promote traditionalist (gender-) norms and values. Because people living in religious contexts are more exposed to religious values in their daily lives than those living in secular contexts, they are more likely to reject homosexuality as a sin. However, our findings also show that in many of Europe’s most religious countries, intolerance toward homosexuals as a group is prevalent too. Looking at Fig 4, we see that almost all of Europe’s most religious countries are located in the South-East. As mentioned above, they all have historical legacies of inter-group conflict, which are often tied to religious identities.

Such cultural legacies have likely contributed to a general tendency toward out-group intolerance, hence homonegativity is more prevalent. This interpretation is plausible and it also concurs with the findings on religious belonging.

A deeper exploration of the influence of historical legacies, nationalism and homonegative political discourses would enhance the knowledge base on homonegativity in Europe further, but lies beyond the scope of this article.

The analysis showed that the three dimensions of religiosity are differently related to the two forms of homonegativity and that the relationships depend on the context.

Religious, socio-economic and cultural national contexts matter for public attitudes toward homosexuality and homosexuals: The populations of highly religious countries, post-soviet countries and countries with high corruption-levels, high income inequality and low levels of gay-rights implementation score significantly higher on both indicators of homonegativity. Future studies monitoring these relationships across Europe should take the multidimensionality of religion and the context-dependence of the relationships into account.

## Conclusion

The results of the analysis reveal that homonegativity is a problem in Europe. In a majority of countries more than half of the population express homonegative attitudes.

However, the analysis showed that not all religiosity is related to both forms of homonegativity. In the majority of European countries traditional believers, although likely to morally reject homosexuality as a sin, are no more likely than non-traditional and non-believers to resent homosexuals as neighbors. People living in Eastern- and South-Eastern European countries, Muslims and Orthodox’ are the most likely to morally reject homosexuality and to resent homosexuals as neighbors. National contexts of high religiosity, high perceived corruption, high inequality and shortcomings in the implementation of gay-rights are strongly linked to homonegativity.

The findings of this article have important implications for European political and religious leaders and policy makers. The findings show how important it is that political and religious leaders carefully reflect what messages they send out in public, in order to avoid spreading intolerance. Policies addressing corruption and income inequality across Europe are likely to indirectly benefit public attitudes toward homosexuals. Policy makers in Europe need to further Europe’s gay rights- agenda and at the same time avoid turning gay-rights policies into a Western-European homonationalist agenda that may be perceived as excluding Europe’s East. Supporting Eastern and South-Eastern European gay-rights activists and making their voices heard locally, as well as on the European level is crucial. It will help make the European gay rights agenda more inclusive, aid in combating homonegativity at the local level and thus promote social peace not only in Western Europe, but also in Europe’s East and South-East.

## Supporting Information

S1 Dataset Supporting Information MLwin-Dataset. Data: EVS 2008; Full MLwin worksheet data-file with model specifications and recodes.
(ZIP)
